# Poor oral health conditions and cognitive decline: Studies in humans and rats

**DOI:** 10.1371/journal.pone.0234659

**Published:** 2020-07-02

**Authors:** Shuang Zhang, Fengchun Yang, Zezheng Wang, Xueshen Qian, Yan Ji, Ling Gong, Song Ge, Fuhua Yan

**Affiliations:** 1 Nanjing Stomatological Hospital, Medical School of Nanjing University, Nanjing, Jiangsu, People’s Republic of China; 2 Hospital of Stomatology, Zunyi Medical University, Zunyi, Guizhou, People’s Republic of China; Istituto Di Ricerche Farmacologiche Mario Negri, ITALY

## Abstract

**Background:**

The relationship between poor oral health conditions and cognitive decline is unclear.

**Objective:**

To examine the association between oral health and cognition in humans and rats.

**Methods:**

In humans: a cross-sectional study was conducted. Cognitive levels were evaluated by the Mini Mental State Examination (MMSE); oral conditions were reflected by the number of missing index teeth, bleeding on probing, and probing pocket depth (PD). In rats: a ligature-induced (Lig) periodontitis model and Aβ_25-35_-induced model of Alzheimer’s disease (AD) were established; tumor necrosis factor-α (TNF-α), interleukin 1 (IL-1), interleukin 6 (IL-6), and C-reactive protein levels in the hippocampus and cerebral cortex were detected.

**Results:**

MMSE scores for the number of missing index teeth ≥ 7 group were significantly lower than those in the ≤ 6 group. A negative relationship (correlation coefficient ρ = −0.310, *P* = 0.002) was observed between MMSE scores and number of missing index teeth. More missing index teeth and lower education levels were independent risk factors for cognitive decline. A negative relationship (correlation coefficient ρ = −0.214, *P* = 0.031) was observed between MMSE scores and average PD. TNF-α and IL-6 levels in the hippocampus of the Lig+AD group were significantly higher than those of the AD group. IL-1 and IL-6 levels in the cerebral cortex of the Lig+AD group were significantly higher than those of the AD group.

**Conclusion:**

Poor oral health conditions including more missing index teeth and higher average PD may be risk factors for cognitive decline. Periodontitis may increase inflammatory cytokines in rat models of AD.

## Introduction

Alzheimer's disease (AD) is an age-related chronic neurodegenerative disorder presenting with progressive cognitive decline with dementia. AD is the most frequent cause of dementia among the elderly [1, 2]. Cognitive decline and AD have a high morbidity and mortality rate, and adversely affect the quality of life among elderly people. The health care costs for patients with AD impinge upon not only their family, but society as a whole [3]. Therefore, the utility of tools to mitigate the negative effects of AD cannot be overstated. In addition to the amyloid hypothesis [4, 5] and tau protein hypothesis [6, 7], inflammation may also play an important role in the pathogenesis of AD [8] and cognitive decline [9].

Periodontitis is a chronic inflammatory disease [10] and a major cause of tooth loss in adults [11]. This disease adversely affects oral health and is associated with systematic inflammatory conditions. Indeed, periodontitis has been linked to more than 50 systemic conditions and diseases [12, 13]. Nevertheless, a precise understanding of the complex relationship between oral health such as periodontitis and cognitive decline is lacking.

Chronic inflammation may lead to neurotoxicity [14–16]. The oral cavity is a major source of exogenous bacteria and peripheral inflammation. Numerous studies, especially those including older adults, have linked tooth loss and oral diseases with decreased cognitive performance, and the onset of dementia and AD [17–19]. However, cognitively impaired individuals may have a reduced capacity to maintain oral health. The temporal sequence and underlying causal associations between cognitive decline and poor oral hygiene need to be clarified. Some studies suggest that poor oral hygiene may lead to cognitive decline [19–21]. However, these studies were limited to questionnaire data, examination of certain oral-related indexes, and serum antibodies to periodontal pathogens. Few studies have assessed the temporal sequence of the process among rats, while also investigating the relationship between oral health and cognition among humans.

Thus, we hypothesized that poor oral hygiene correlates with, and may even lead to, cognitive decline. The aim of this study was to explore whether poor oral health conditions indeed have a relationship with, or lead to cognitive decline in humans and rats.

## Materials and methods

### Study population

This cross-sectional study was conducted in Nanjing, Jiangsu, China. The individuals included in the present study were recruited between 2016 and 2018. All medical records were collected between 2016 and 2018, and the study was conducted in 2019. Authors had no access to information that could identify individual participants during or after data collection. In total, 102 individuals aged 52 to 101 years were enrolled. The participants were asked to complete Mini-Mental State Examination (MMSE) questionnaires and to undergo dental examinations performed by specially trained professionals (the selection criteria and training course for those professionals are outlined in S1 Fig). The participants were also asked to provide information concerning their sexes, ages (in years), education levels, and any histories of hypertension, diabetes, cardiovascular diseases, hyperlipidemia, stroke events, cerebral trauma, or smoking. If participants were unable to recall this information, the self-statement form was filled in by their guardians. The sample sizes were 39 for the 7–10 teeth-lost group, and 63 for the 0–6 teeth-lost group. The sample size was calculated according to the method described by Chow et al. [22]. A total sample size of 62 participants (31 participants with 0–6 missing index teeth and 31 with 7–10 missing index teeth), at a two-tailed significance level of 0.05, could provide statistical power of 80% to detect the differences between the cognitive scores of the two groups. The mean ± standard deviation (SD) used in calculation was 27.143 ± 2.632 and 24.111 ± 5.302, respectively. We calculated the mean ± SD from a preliminary experiment.

### Dental examinations

All participants underwent three types of clinical dental examinations, which included counting the number of missing index teeth, observing for bleeding on probing (BOP) with the remaining index teeth, and measuring the value of probing pocket depth (PD) of the remaining index teeth. The index teeth were teeth labelled 11, 16, 17, 26, 27, 31, 36, 37, 46, and 47 in accordance with the index teeth of the community periodontal index (CPI) [23–25]. These are the 10 teeth labelled in red in S2 Fig. The World Health Organization (WHO) has provided evidence for the validity of using these index teeth [23, 24].

All trained professionals started the examination by performing a visual inspection. They counted the number of missing index teeth with the help of sterile single-use dental instruments (odontoscope, CPI probe and dental tweezer) (Nanjing Sealand Medical, Nanjing, China). The index teeth that were not visible clinically were judged to be missing.

To assess BOP and PD of the participants’ remaining index teeth, CPI probes (Nanjing Sealand Medical, Nanjing, China) were used to examine the six conventional sites (mesio-buccal, mid-buccal, disto-buccal, disto-lingual, mid-lingual, and mesio-lingual) of each tooth as per previous recommendations [26–28]. CPI probes are recommended by the WHO to identify BOP and measure PD for individuals over 35 years of age during epidemiological studies [29, 30]. The CPI probe has a small 0.5-mm ball tip that helps detect calculus and minimizes penetration into the soft tissues, thereby reducing discomfort [24, 26]. Two black bands on the CPI probe denote the lengths, 3.5 mm to 5.5 mm, and 8.5 mm to 11.5 mm, respectively [24, 26]. The evaluation of PD and BOP were performed in accordance with the recommendations of the WHO (2013) and previous studies [31–34]. The CPI probe was gently placed on the sites of the teeth to be examined, parallel to the long axis of the teeth and moved slowly up and down following the tooth anatomy, with a force of approximately 20 g at the tip of the probe [35]. PD was defined as the distance between the free gingival margin and the bottom of the pocket/gingival sulcus and was recorded in millimeters [34]. The maximum value for all six sites was identified as the representative PD of that tooth [24, 30]. The representative PD was recorded as level 0, 1, and 2 for PD ≤ 3 mm, 3 < PD < 6 mm, and PD ≥ 6 mm, respectively. The PD of missing teeth was recorded as level 3 to exclude its influence on the MMSE scores. The average PD level was calculated for each individual. BOP was considered positive if bleeding occurred within 30 s following the insertion and removal of the CPI probe, and was recorded as BOP (+) [35]. BOP (–) denoted no bleeding on probing. If there was at least one site among the six sites that showed positive bleeding on probing, we recorded the tooth as BOP (+).

### Cognitive testing

The MMSE questionnaire, which is recommended by the American Academy of Neurology Guidelines, for the clinical assessment of the degree of cognitive impairment, with a score ranging from 0 to 30 points, was used to evaluate the cognitive state of the participants (higher scores indicated better cognitive function) [6, 36].

### Animal models

We made every attempt to alleviate suffering in the following experiments with animal subjects, including using as few rats as possible, providing anesthesia before procedures that may cause pain, performing surgery or euthanasia after confirming the effectiveness of anesthesia, and completing the procedure or sacrifice before the anesthetic wore off. Male Sprague-Dawley rats (n = 24, 12 weeks old, 220 ± 30 g) were purchased from the Experimental Animal Center of Third Military Medical University (clean-grade, Certificate NO. SCXK 2012–0005). Rats were group-housed in a clean-grade animal room (room temperature, 23 ± 1°C; relative humidity maintained at 60%) on a 12/12-hour light/dark cycle.

After 1 week of adaptive feeding, the rats were randomly divided into four groups (n = 6 per group): control, ligature-induced (Lig), AD, and Lig+AD. Control and AD groups were provided with *ad libitum* access to normal food and water. For control and AD groups, oral cleaning and maintenance were performed once a week. Rats in the Lig and Lig+AD groups were subjected to ligation of maxillary bilateral second molars with silk thread and 0.02 mm steel wires while under anesthesia. Rats were anesthetized with an intraperitoneal injection of 2% (w/v) sodium pentobarbital (0.3 mL/100 g body weight) (Lan Tai Chem, Beijing, China). Rats in Lig and Lig+AD groups were provided water and food immersed for 8 hours in 10% sucrose *ad libitum*.

After 6 weeks of ligation, Aβ-induced cognitive impairments were induced in rats as described previously [37]. Aβ_25−35_ (Sigma-Aldrich, St. Louis, USA) was dissolved in sterilized normal saline to a final concentration of 2 μg/μL. The rats in AD and Lig+AD groups were anesthetized using the above-mentioned method and fixed in a stereotaxic apparatus. After the head was shaved and sterilized, a drilling procedure was applied at a point 4 mm behind the anterior fontanel and 2.5 mm from the midline. After breaking through the skull, a microinjection needle was lowered to 3.5 mm below the skull. A sporadic AD animal model was implemented by injecting 5 μL of prepared Aβ_25–35_ per hemisphere into the bilateral hippocampus over 5 minutes. The needle was slowly removed after 4 minutes. After injection, the wound was sutured with 3–0 silk thread. Rats were maintained on respective diets for 13 days as described above.

### Morris Water Maze (MWM)

The MWM test was used to evaluate spatial memory and learning, consistent with previous research [38–40]. Trials were conducted daily over 14–18 days following the injection of Aβ_25–35_. The test consisted of a black circular pool (160 cm in diameter and 50 cm in height) and a black platform (12 cm in diameter and 2 cm below the water surface). The depth of water was approximately 30 cm. The pool was divided into four quadrants. The platform was located in the third quadrant. The experiment was conducted over 5 days. The experimental results were recorded by TopScan (CleverSys, Reston, VA, USA) behavioral analysis system. All experiments were conducted in the dark. The temperature of the water was controlled at 23 ± 2°C. The test was divided into two stages. First, rats were individually placed into the water from the fixed entry points of quadrants 1, 2, and 4 facing the wall of the pool. The time taken to find the platform within 120 s was recorded once a day. The average time recorded in the three quadrants was taken as the average escape latency on the same day in this navigational experiment. If the platform was not found within 120 s, 120 s was recorded as the escape latency, and the rats were guided to and positioned on the platform for 15 s. The first stage lasted for 4 consecutive days and started at 19:00 every day. The second stage was started on the fifth day. In this stage, the platform was removed, and rats were placed into the pool from the first quadrant entry point. Swimming speed and number of times of crossing the target quadrant were recorded.

#### Tissue preparation

After the MWM test, three rats were randomly selected from each group. The selected rats were sacrificed using the following method. They were first anesthetized using the method mentioned above. Subsequently, they were placed in the supine position, and fixed by 4% paraformaldehyde cardiopulmonary perfusion. The perfusion was stopped after the whole body was stiff and the brain tissue was fully fixed. Whole brains were removed and fixed in 4% paraformaldehyde for 1 week and then embedded in paraffin.

The remaining rats were sacrificed using excessive intraperitoneal injection of sodium pentobarbital. The bilateral hippocampus and cerebral cortex were immediately separated; extreme care was taken due to the brittle nature of these structures. The hippocampus and cerebral cortex were stored at −80°C until enzyme-linked immunosorbent assay (ELISA) analysis. The maxillary alveolar bones of rats were collected and divided into two parts along the midline of the palate and were used to observe the degree of alveolar bone absorption.

### Gross observation of alveolar bone

The gingiva and surrounding soft tissues were removed from the maxillae of the rats. The residual maxillae were soaked in 3% hydrogen peroxide solution for 12 hours, washed in phosphate-buffered saline (PBS) three times, dried, stained with 1% methylene blue dye, and washed with PBS immediately. Photos were obtained using a digital camera (ILCE-5100L, Sony, Japan).

### Hematoxylin and eosin (H&E) staining

The fixed brain tissues were embedded in paraffin. After coronal sectioning (3 μm sections) with a slicer (RM2245, Leica, Germany), H&E staining was performed with an ST5020 automatic H&E dyeing machine (Leica, Germany). Histopathological changes were observed with optical microscopy (KS300, Zeiss-Kontron, Germany).

### ELISA assay

In each group, 50 mg of hippocampal tissue was homogenized in 500 μL PBS (pH 7.4) with 5 μL protease inhibitors and 70 mg of cerebral cortical tissue was treated in the same manner. The homogenates were placed on ice for 10 minutes and then centrifuged (4°C, 12,000 revolutions per minute, 10 minutes). The supernatant was collected, and protein concentration was determined using a bicinchoninic acid protein analysis kit (Beijing Solarbio Science & Technology, Beijing, China). The concentrations of Aβ_1–40_, tumor necrosis factor-α (TNF-α), interleukin 1 (IL-1), interleukin 6 (IL-6), and C-reactive protein (CRP) in the hippocampus and the cerebral cortex were detected using ELISA kits (Shanghai Jianglai Biotechnology Co., Ltd., China).

### Ethics statement

This study was conducted in accordance with all ethical standards including ethics committee approval and consent procedures. The procedures of this study involving experiments on human subjects were carried out in accord with the recommendations of the National Commission for the Protection of Human Subjects of Biomedical and Behavioral Research and with the Helsinki Declaration of 1975. The studies involving human participants were reviewed and approved by China Oral Health Foundation. The participants and their guardians provided their written informed consent prior to participation. The procedures of this study involving experiments on animal subjects were done in accord with the National Research Council's guide for the care and use of laboratory animals. Animal experiments were performed in accordance with the State Committee of Science and Technology of People’s Republic of China Order No. 2 in November 1988 (revised in 2013). The animal study was reviewed and approved by the Experimental Animal Ethics Committee of Zunyi Medical University.

### Statistical analyses

All analyses were conducted in the IBM SPSS Statistics for Windows (Version 24.0). A two-tailed probability of < 0.05 was considered significant for all tests. Descriptive statistics for certain characteristics were calculated and these data are shown as the number and percent of cases, n (%), or the mean and 95% confidence interval (CI). Independent sample t-tests were used to compare the significance of differences between continuous variable means when only two groups were compared. Continuous variable means of more than two groups were compared using a one-way analysis of variance (ANOVA) followed by the least significant difference (equal variance) or Dunnett’s T3 (unequal variance) post-hoc test. We used multiple linear regression and stepwise method to analyze the relationship between MMSE scores and potential factors. Based on past research [41, 42], we selected the following independent variables: sex, age (years), education level, hypertension, diabetes, cardiovascular diseases, hyperlipidemia, prior stroke events, cerebral trauma, smoking, number of missing index teeth, and grouped number of missing index teeth (i.e., 0–6 and 7–10). The dependent variable was MMSE score. Spearman rank correlation was used to analyze monotonic associations for ordinal data [43].

## Results

### Human subjects

Descriptive characteristics, mean MMSE scores, and *P*-values for human participants. The results for humans are based on 102 individuals (69 female) and 1,020 teeth (514 missing). The age distribution in the different cohorts were as follows: 13 in the young old cohort (52–66 years), 22 in the old cohort (67–81 years), and 67 in the old-old cohort (82–101 years). The n (%) of the population, mean MMSE scores, and *P*-values for age, sex, and years of education are shown in Table 1. Medical and smoking histories are shown in Table 2.

**Table 1:** Participants’ information related to age, sex, and education level.

Characteristics	n^a^ (%)	Mean MMSE^b^ scores (95% CI^c^)	*P*-Values^d^
Age group			
52–66 (A)	13 (12.7)	26.8 (24.6–28.9)	A vs. B 0.313
67–81 (B)	22 (21.6)	24.1 (20.7–27.4)	B vs. C 0.337
82–101 (C)	67 (65.7)	22.3 (20.3–24.3)	A vs. C 0.054
Sex			
Female	69 (67.6)	22.7 (20.7–24.7)	0.197
Male	33 (32.4)	24.5 (22.4–26.6)	
Education (years)			
<1	16 (15.7)	13.0 (8.1–17.9)	
1–5	1 (1.0)	29	
6–8	8 (7.8)	22.5 (16.1–28.9)	
9–11	26 (25.5)	23.0 (20.6–25.5)	
12–15	22 (21.6)	26.5 (24.5–28.4)	
>16	29 (28.4)	26.7 (24.9–28.4)	

^a^The number of cases

^b^Mini Mental State Examination

^c^Confidence interval

^d^Independent sample t-tests

**Table 2:** Participants’ information based on medical history.

Characteristics	n^a^ (%)	Mean MMSE^b^ scores (95% CI^c^)	*P* Values^d^
**Medical history**			
Hypertension			
Yes	49 (48.0)	24.2 (22.3–26.2)	0.210
No	53 (52.0)	22.3 (20.1–24.6)	
Diabetes			
Yes	26 (25.5)	23.5 (20.7–26.3)	0.851
No	76 (74.5)	23.2 (21.4–25.0)	
Cardiovascular diseases			
Yes	32 (31.4)	24.8 (22.5–27.0)	0.183
No	70 (68.6)	22.6 (20.6–24.5)	
Hyperlipidemia			
Yes	17 (16.7)	26.0 (23.0–29.0)	0.105
No	85 (83.3)	22.7 (21.0–24.4)	
Cerebral trauma			
Yes	2 (2.0)	23.0 (−53.2–99.2)	0.962
No	100 (98.0)	23.3 (21.7–24.8)	
Prior stroke			
Yes	21 (20.6)	22.4 (19.0–25.8)	0.559
No	81 (79.4)	23.5(21.8–25.2)	
Smoking			
Active smokers	9 (8.8)	21.7 (16.8–26.6)	0.516
Former or non-smokers	93 (91.2)	23.4 (21.8–25.0)	

^a^The number of cases

^b^Mini Mental State Examination

^c^Confidence interval

^d^Independent sample t-tests

#### Number of missing index teeth and cognitive scores

**Fig 1:**
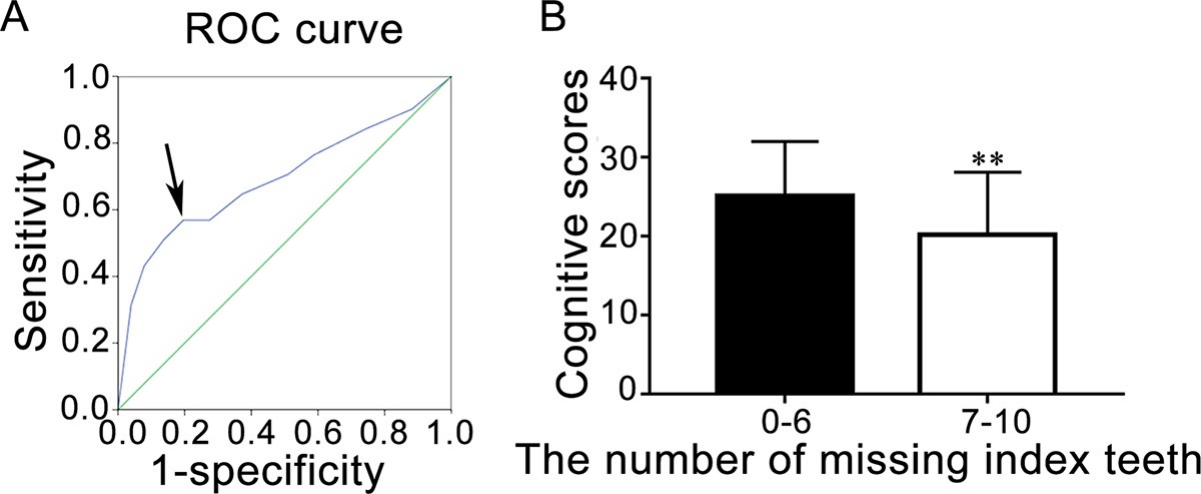
Number of missing index teeth and cognitive scores. (A) ROC curve. The arrow indicates the cut-off point for grouping of the number of missing index teeth. (B) Grouping of the number of missing index teeth and cognitive scores. Data are presented as mean ± SD (n = 102); ** *P* < 0.01, vs. 0–6, Student’s t-test.

We present here only those *P***-**values for where significant differences in the number of missing index teeth were observed. The arrow in the receiver operating characteristic (ROC) curve (Fig 1A) indicates that the point of the number of missing index teeth is 6.5 (closest to the top left corner). This point represents the optimal compromise between specificity and sensitivity [44]. According to the ROC curve, we grouped the number of missing index teeth as 0–6 and 7–10. Independent t-test of the two groups and cognitive scores resulted in a *P***-**value of 0.001. This indicates that, compared with the number of missing index teeth ≤ 6 (cognitive score [mean ± SD] = 25.16 ± 6.84), the number of missing index teeth ≥ 7 (cognitive score [mean ± SD] = 20.18 ± 7.93) was associated with lower cognitive scores (Fig 1B).

#### Multivariate analyses of the number of missing index teeth

Spearman rank correlation revealed a weak negative association (correlation coefficient ρ = −0.310, *P* = 0.002) between MMSE scores and the number of missing index teeth. To further explore the relationship between missing teeth and MMSE scores, a multiple linear regression analysis was conducted. The multivariate analysis (stepwise multiple linear regression) with the highest coefficient of determination (adjusted R^2^ = 0.370) included the following variables: number of missing index teeth (*P* = 0.008) and education level (*P* < 0.001). These two indicators were significantly associated with MMSE results. Sex, age (years), hypertension, diabetes, cardiovascular diseases, hyperlipidemia, prior stroke events, cerebral trauma, smoking, and grouped missing index teeth did not significantly influence MMSE scores independently. This regression analysis indicated that among the characteristics we considered, the factor with the second highest influence on MMSE scores was the number of missing index teeth. In summary, the regression model of number of missing index teeth and education level produced the following equation:

$$f(x)=a_{0}+0.545a_{1}-0.218a_{2}$$

In the equation above, *f*(*x*) represented MMSE scores, *a*_0_ was 15.261, *a*_1_ represented education level, and *a*_2_ represented the number of missing index teeth. Taking potential influencing factors into account, stepwise multiple linear regression analysis still identified the number of missing index teeth as an independent variable for MMSE scores.

#### Periodontal-related indices

BOP (+) was observed in 224 of 475 teeth (47.2%). The average MMSE score of BOP (+) teeth is 24.8 (95% CI, 24.0–25.7), which was lower than that of BOP (–) teeth with an average MMSE score of 25.3 (95% CI, 24.4–26.1). Independent sample t-tests revealed no significant differences between the MMSE scores of the two groups (*P* = 0.494) (Table 3).

**Table 3:** Dental examinations for missing index teeth, BOP^a^, and PD^b^.

Characteristics	n^c^ (%)	Mean MMSE^d^ scores (95% CI^e^)	*P*-Values
Number of missing index teeth			
0	11 (10.8)	24.2 (20.4–28.0)	(0 vs. 9) 0.045^f^
1	10 (9.8)	27.4 (25.3–29.5)	(1 vs. 9) 0.004^g^
2	12 (11.8)	24.1 (18.4–29.8)	(1 vs. 10) 0.008^g^
3	7 (6.9)	24.6 (18.0–31.1)	(2 vs. 9) 0.044^f^
4	10 (9.8)	26.3 (22.8–29.8)	(4 vs. 9) 0.011^f^
5	9 (8.8)	22.8 (14.6–30.9)	(4 vs. 10) 0.021^f^
6	4 (3.9)	29.0 (26.8–31.3)	(6 vs. 9) 0.010^f^
7	6 (5.9)	24.3 (17.2–31.5)	(6 vs. 10) 0.021^f^
8	7 (6.9)	21.7 (16.4–27.1)	
9	8 (7.8)	17.3 (8.2–26.3)	
10	18 (17.6)	19.5 (15.8–23.2)	
BOP			
Yes	224 (47.2)	24.8 (24.0–25.7)	0.494^h^
No	251 (52.8)	25.3 (24.4–26.1)	
PD levels			
0^i^	222 (22.4)	24.8 (23.9–25.7)	(0 vs. 3) < 0.001^m^
1^j^	235 (23.7)	25.3 (24.4–26.1)	(1 vs. 3) < 0.001^m^
2^k^	18 (1.8)	26.1 (23.5–28.8)	(2 vs. 3) 0.014^f^
3^l^	515 (52.0)	21.6 (20.9–22.3)	

^a^Bleeding on probing

^b^Probing pocket depth

^c^The number of cases

^d^Mini Mental State Examination

^e^Confidence interval

^f^*P* < 0.05; one-way analysis of variance

^*g*^*P* < 0.01; one-way analysis of variance

^h^Independent sample t-tests

^i^PD ≤ 3 mm

^j^3 < PD < 6 mm

^k^PD ≥ 6 mm

^l^PD of missing index teeth

^m^*P* < 0.001; One-way analysis of variance.

The PD of 475 teeth was measured (Table 3). We grouped PD ≤ 3 mm as level 0, 3 < PD < 6 mm as level 1, and PD ≥ 6 mm as level 2. The PD of missing index teeth was classified as level 3. There were 222 (22.4%), 235 (23.7%), 18 (1.8%), and 515 (52.0%) teeth in level 0, 1, 2, and 3, respectively.

#### Correlation between PD and MMSE scores

Spearman rank correlation revealed a negative relationship (correlation coefficient ρ = −0.214, *P* = 0.031) between MMSE scores and average PD of the individuals. The multiple linear regression did not include the average PD in the equation.

### Rat model

#### Periodontitis rat model

**Fig 2:**
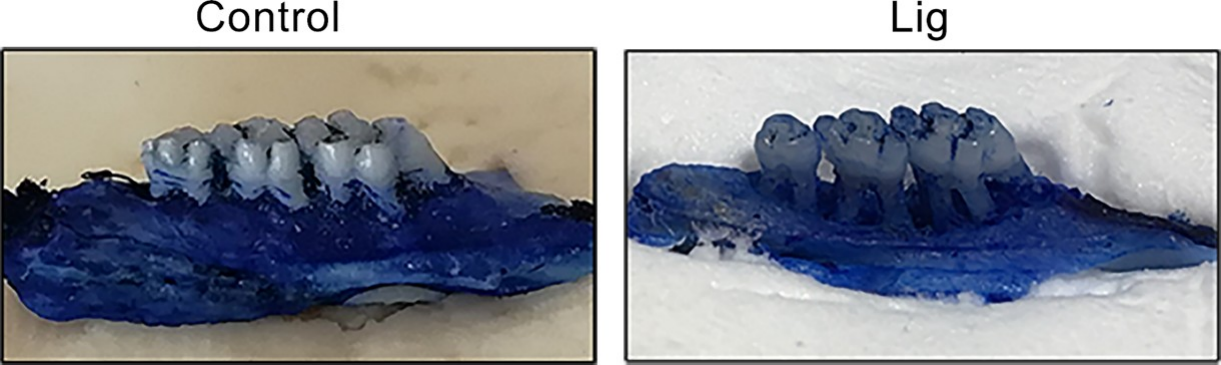
Gross observation of ligation and high-sugar soft food induced periodontitis in rats (n = 6).

The periodontal tissues of the maxillary second molars in the Lig group were severely damaged, and the alveolar bone was absorbed to one-third of the apex. No significant abnormality was observed in the control group (Fig 2). This indicated that the periodontitis model was successfully established.

**Fig 3:**
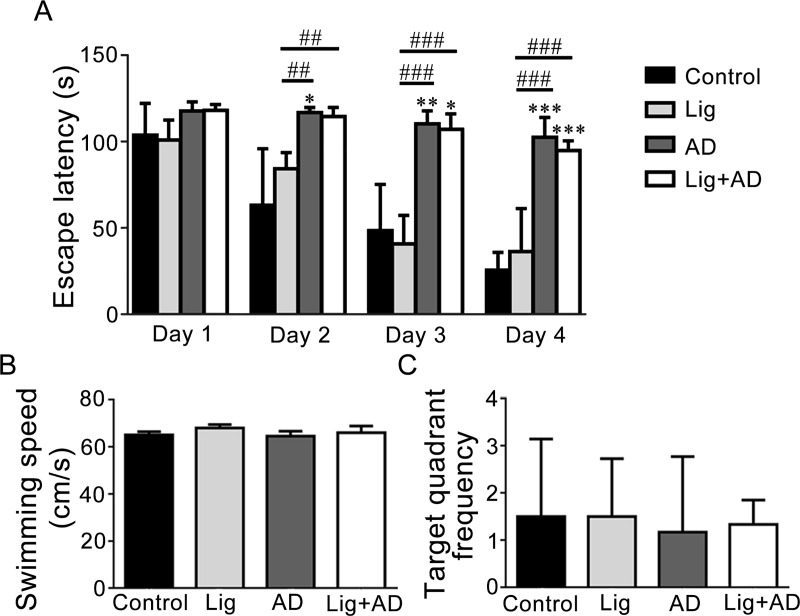
MWM results. (A) Escape latency to reach the hidden platform from days 1 to 4. (B) Swimming speed of the rats. (C) Target quadrant crossover frequency. Data are presented as mean ± SD (n = 6). *P < 0.05, **P < 0.01, ***P < 0.001 vs. control, ##*P* < 0.01, ###*P* < 0.001 vs. other groups, one-way ANOVA.

Effects of periodontitis on Aβ_25–35_ induced spatial learning and memory impairment. MWM was used to examine memory function. As shown in Fig 3A, the escape latency of rats in each group decreased from day 1 to day 4, indicating learning had occurred over 4 days of swimming training. From the third day onward, the escape latencies of the AD and Lig+AD groups were significantly longer than that of the control group, indicating poorer learning and memory. This suggests that injection of Aβ_25–35_ into the hippocampus led to a decline in learning and memory in rats. However, the differences in escape latency between the Lig+AD and AD groups were not significant. The recorded swimming speeds (Fig 3B) were not significantly different between the groups, indicating that different escape latencies were not induced by different swimming speeds. The number of times that each rat crossed the target quadrant was not significantly different between groups (Fig 3C).

**Fig 4:**
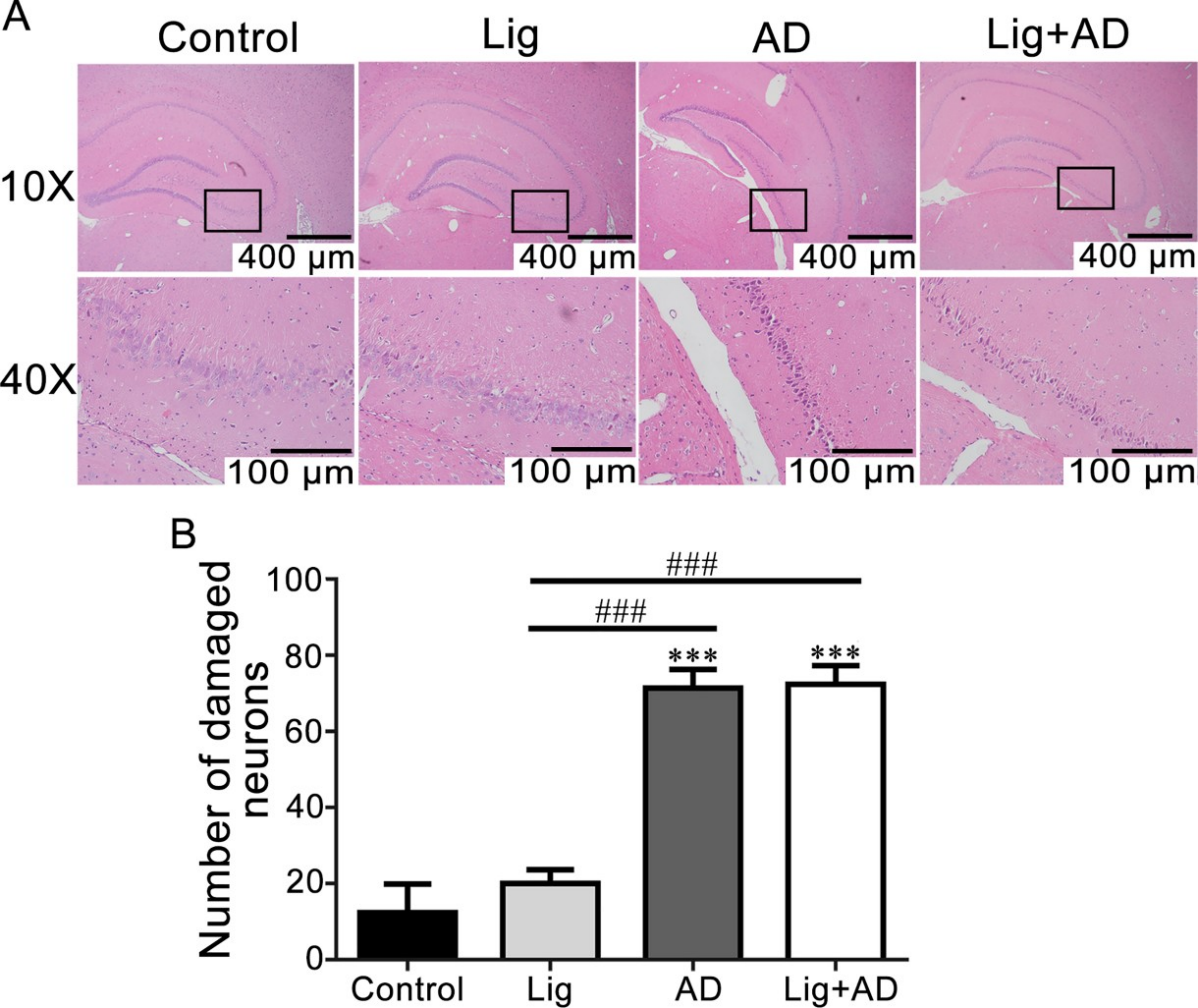
Cellular morphological alterations and neuronal damage in the CA3 region of the hippocampus. (A) H&E staining images of hippocampal CA3 region. (B) Quantitative analysis of neuronal damage in hippocampal CA3 region. Data are presented as mean ± SD (n = 6). ***P < 0.001 vs. control, ###*P* < 0.001 vs. other groups, one-way ANOVA.

Effects of periodontitis on Aβ_25–35_ induced neuronal damage and morphological abnormalities. H&E-stained sections of brain tissues were observed by optical microscopy. Hippocampal neurons were observed, and the number of damaged neurons was counted. The number of hippocampal neurons in the control and Lig groups was higher, the structures were more complete, and the nucleoli were more evident than those in AD and Lig+AD groups (Fig 4A). The number of damaged neurons in the CA3 area of the hippocampus in the AD and Lig+AD groups was significantly higher than that in the control and Lig groups (Fig 4B). In the AD and Lig+AD groups, the layers of neurons were reduced. Some cells showed deep staining, karyopyknosis, and increased cell gaps.

#### Effects of periodontitis on the formation of Aβ_1–40_ in brain tissue of rats with AD

**Fig 5:**
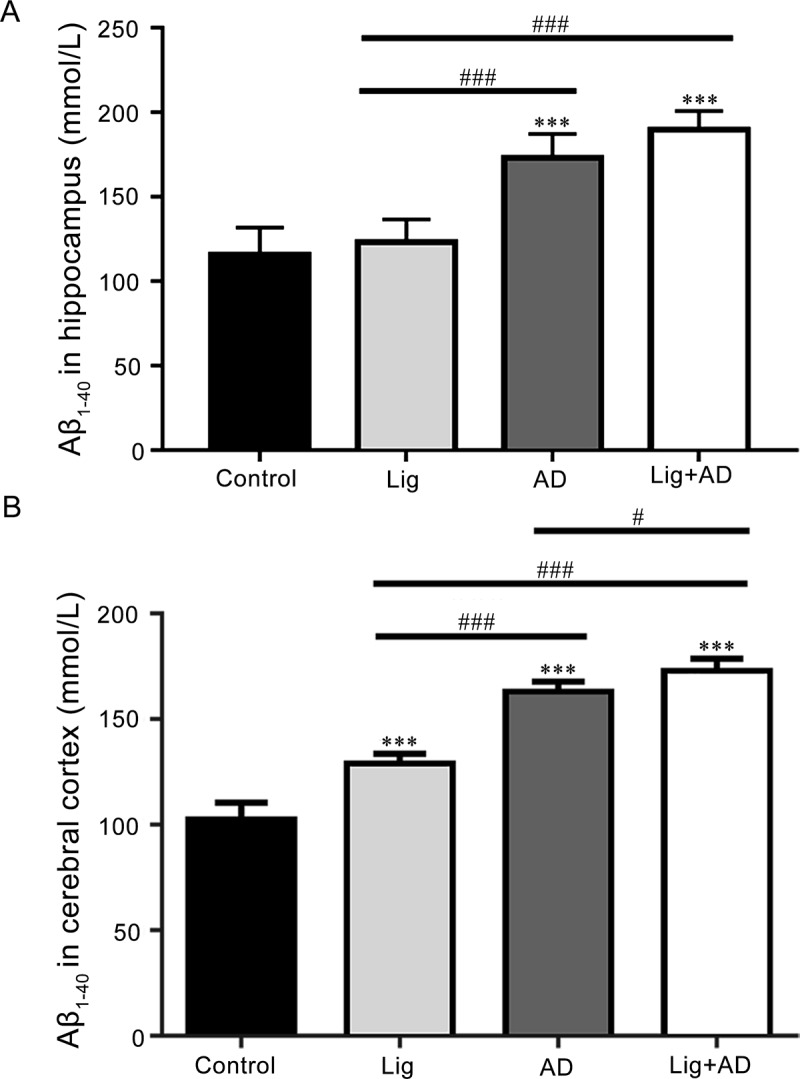
Concentrations of Aβ_1–40_ in the hippocampus and the cerebral cortex. Concentrations of Aβ_1–40_ in the (A) hippocampus; (B) cerebral cortex. Data are presented as mean ± SD (n = 6). ***P < 0.001 vs. control, #*P* < 0.05, ###*P* < 0.001 vs. other groups, one-way ANOVA.

Aβ_1–40_ concentrations in the hippocampus (Fig 5A) and cerebral cortex (Fig 5B) of the AD and Lig+AD groups were significantly higher than those of the control and Lig groups. Aβ_1–40_ concentration in the cerebral cortex of the Lig+AD group was significantly higher than that of the AD group (Fig 5B).

**Fig 6:**
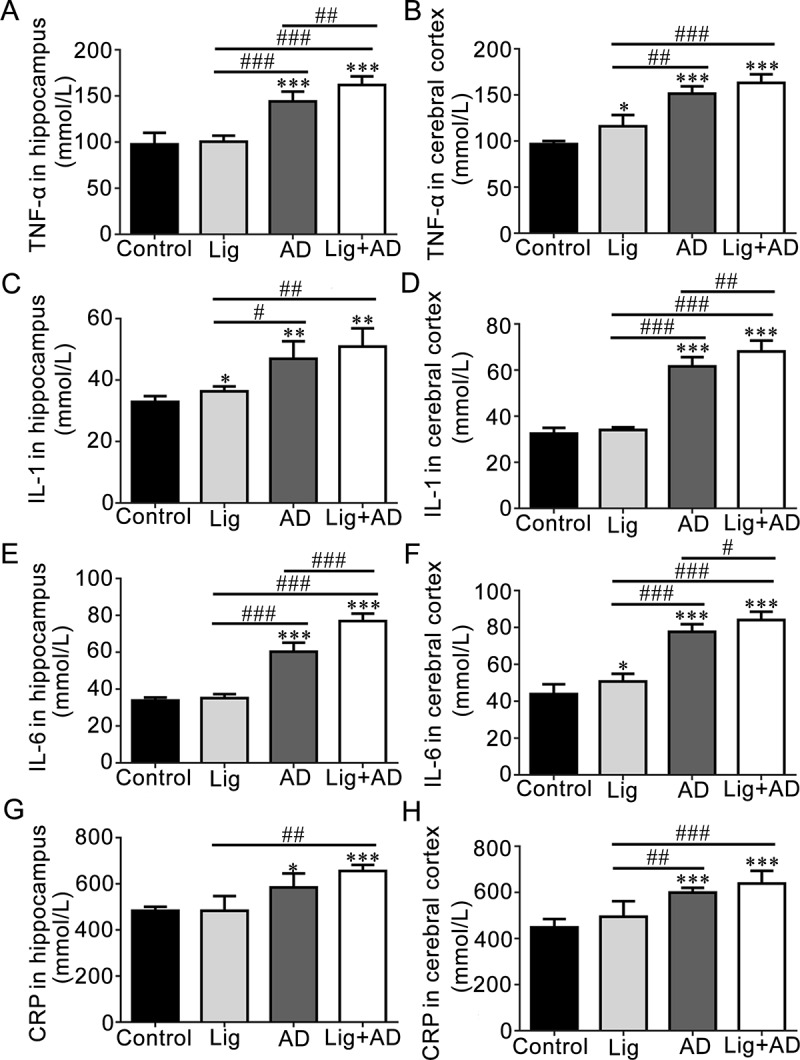
Concentrations of TNF-α, IL-1, IL-6, and CRP in the hippocampus and cerebral cortex. Concentrations of (A) TNF-α in hippocampus. (B) TNF-α in cerebral cortex. (C) IL-1 in hippocampus. (D) IL-1 in cerebral cortex. (E) IL-6 in hippocampus. (F) IL-6 in cerebral cortex. (G) CRP in hippocampus. (H) CRP in cerebral cortex. Data are presented as mean ± SD (n = 6). *P < 0.05, **P < 0.01, ***P < 0.001 vs. control, #*P* < 0.05, ##*P* < 0.01, ###*P* < 0.001 vs. other groups, one-way ANOVA.

Concentrations of TNF-α in the hippocampus and cerebral cortex of rats with AD and periodontitis. Concentrations of TNF-α in the hippocampus (Fig 6A) and cerebral cortex (Fig 6B) of the AD and Lig+AD groups were significantly higher than those of the control groups. TNF-α levels in the hippocampus (Fig 6A) of the Lig+AD group were significantly higher than those of the AD group.

Concentrations of IL-1 in the hippocampus and cerebral cortex of rats with AD and periodontitis. IL-1 levels in the hippocampus (Fig 6C) and cerebral cortex (Fig 6D) of the AD and Lig+AD groups were significantly higher than those of the control group. IL-1 levels in the cerebral cortex (Fig 6D) of the Lig+AD group were significantly higher than those of the AD group.

#### Concentrations of IL-6 in the hippocampus and cerebral cortex of rats with AD and periodontitis

IL-6 levels in the hippocampus (Fig 6E) and cerebral cortex (Fig 6F) of the AD and Lig+AD groups were significantly higher than those of the control groups. IL-6 levels in the hippocampus (Fig 6E) and the cerebral cortex (Fig 6F) of the Lig+AD groups were significantly higher than those of the AD groups.

Concentrations of CRP in the hippocampus and cerebral cortex of rats with AD and periodontitis. CRP levels in the hippocampus (Fig 6G) and cerebral cortex (Fig 6H) of the AD and Lig+AD groups were significantly higher than those of the control group.

## Discussion

In the human population we investigated, ≥ 7 missing index teeth were associated with lower cognitive scores. A higher number of missing index teeth was a risk factor for lower MMSE scores. There was a negative relationship between MMSE scores and average PD. Results from our rat models indicate that, compared with those of the AD group, IL-1, IL-6, and TNF-α concentrations of the Lig+AD group were significantly higher. This suggested that periodontitis before AD increases inflammatory cytokines.

Neuropsychological functions were evaluated using MMSE. One previous study suggested that the Montreal Cognitive Assessment (MoCA) test meets the criteria better than the MMSE for the detection of mild cognitive impairment among the population aged over 60 years old [45]. We employed the MoCA test in 41 of our volunteers, but most of them complained about the long investigation time. The MMSE is a brief screening test that can document cognitive changes and quantitatively assess the level of cognitive impairment [46]. Among the short screening tools for the measurement of cognitive impairment in community and research settings, the MMSE is the best known and the most commonly used test [47]. The MMSE saves time and seldom causes impatience among the surveyed population; for studies with similar aims to ours, the MMSE is recommended.

The area under the ROC curve (sensitivity and 1−specificity) is often evaluated to facilitate decision-making [44]. The point closest to the top left presented an appropriate cut-off, affecting the sensitivity and specificity of the test [44]. At this point in our study, the number of missing index teeth was 6.5; therefore, we selected 6 as the grouping criterion. The number of missing index teeth ≥ 7 was associated with lower cognitive scores. Tooth loss reflects poor oral health status and reduces chewing ability. Missing teeth [20] and the reduction in the masticatory ability [48, 49] can affect cognition, which is consistent with our results.

Periodontitis and AD models in rats were used to further investigate the possible mechanisms. The Aβ_1–40_ concentration in the cerebral cortex of the Lig+AD group of rats was significantly higher than that of the AD group, which suggests that periodontitis might promote the deposition of Aβ in the brain. Concentrations of TNF-α, IL-1, IL-6, and CRP in the hippocampus and cerebral cortex of the AD and Lig+AD groups were significantly higher than those of the control groups, which suggests that injection of Aβ_25–35_ into the hippocampus may increase the concentrations of these inflammatory cytokines in the hippocampus and cerebral cortex of rats.

Cognitively impaired populations have been identified as useful targets for identifying preclinical AD [6]. In our periodontitis and AD rat models, the levels of TNF-α and IL-6 in the hippocampus, and IL-1 and IL-6 in the cerebral cortex of the Lig+AD group were significantly higher than those of the AD group. These results suggest that periodontitis may increase the concentrations of some proinflammatory cytokines in the hippocampus and cerebral cortex of rats, which fits with previous research [50]. Proinflammatory cytokines IL-1, IL-6, and TNF-α are markers of inflammation [51]. Elevated levels of certain inflammatory cytokines, such as IL-1, IL-6, and TNF-α are associated with an increased risk of AD [52, 53]. Inflammatory cytokines may be one of the mediators by which poor oral health conditions lead to cognitive decline. In this manner, many pro-inflammatory cytokines circulate in systemic tissues, such as the hippocampus and cerebral cortex. Studies have reported a more direct way by which poor oral health conditions may lead to cognitive decline. The keystone pathogen in chronic periodontitis, *Porphyromonas gingivalis*, has been identified in the brains of AD patients [54, 55]. Small-molecule inhibitors targeting gingipains have been shown to reduce neuroinflammation and rescue neurons in the hippocampus [54].

In this study, only the periodontitis model did not affect the memory test and histopathological study. However, it did affect the tests for inflammatory cytokines; for example, Aβ_1–40_ in the cerebral cortex (P < 0.001), TNF-α and IL-6 in the cerebral cortex (P < 0.05), and IL-1 in the hippocampus (P < 0.05).

The maintenance of oral health may be a new frontier in cognitive preservation among the aging population. Good oral hygiene, treatment of periodontitis, and maintenance of overall oral health may all be useful treatment strategies for the conservation of optimal neuronal function. This approach may advance the understanding, prevention, and management of aging-associated conditions. Non-pharmaceutical interventions, such as proper brushing and flossing, cleaning teeth regularly, curing periodontitis, and maintaining teeth are relatively simple, inexpensive, and non-invasive approaches for delaying cognitive decline and AD.

Several limitations of this study should be noted. First, the human participants were grouped in general categories without considering subdivisions, such as oral health, gingivitis, and periodontitis, since the sample size was relatively small. Second, for ethical reasons, we did not collect blood samples and imaging data of the volunteers. Blood samples and imaging would allow a more accurate analysis of oral health conditions and cognitive state of the participants. Third, we established AD and Lig models in rats but did not detect periodontitis pathogens in the brain. Further studies with a larger sample size and more extensive examinations are needed to validate our initial observations and claims. The results of this study highlight the maintenance of oral health as one potential therapeutic approach to prevent cognitive decline.

This study demonstrates that periodontitis is a relatively easy to control peripheral chronic inflammation and a risk factor for AD. Our results may facilitate the development of novel preventative approaches and therapies to slow cognitive decline in elderly and AD populations.

## Supporting information

S1 Checklist (DOCX)

S1 Fig Training and evaluation program for the trained professionals. (TIF)

S2 Fig Index teeth according to CPI. The numbers assigned to teeth are their specified names in dentistry. The 10 teeth labelled in red are the index teeth according to the Community Periodontal Index.
(TIF)

## References

[1] Moretti DV, Frisoni GB, Binetti G, Zanetti O. Comparison of the effects of transdermal and oral rivastigmine on cognitive function and EEG markers in patients with Alzheimer's disease. Front Aging Neurosci. 2014;6:179 10.3389/fnagi.2014.00179 25100996 PMC4107674

[2] Lang V, Zille M, Infante-Duarte C, Jarius S, Jahn H, Paul F, et al Alzheimer's disease: Elevated pigment epithelium-derived factor in the cerebrospinal fluid is mostly of systemic origin. J Neurol Sci. 2017;375:123–128. 10.1016/j.jns.2017.01.051 28320113

[3] Naorungroj S, Schoenbach VJ, Beck J, Mosley TH, Gottesman RF, Alonso A, et al Cross-sectional associations of oral health measures with cognitive function in late middle-aged adults: a community-based study. J Am Dent Assoc. 2013;144(12):1362–1371. 10.14219/jada.archive.2013.0072 24282266 PMC4955404

[4] Hardy J, Selkoe DJ. The amyloid hypothesis of Alzheimer's disease: progress and problems on the road to therapeutics. Science. 2002;297(5580):353–356. 10.1126/science.1072994 12130773

[5] Karran E, Mercken M, De Strooper B. The amyloid cascade hypothesis for Alzheimer's disease: an appraisal for the development of therapeutics. Nat Rev Drug Discov. 2011;10(9):698–712. 10.1038/nrd3505 21852788

[6] Scheltens P, Blennow K, Breteler MM, de Strooper B, Frisoni GB, Salloway S, et al Alzheimer's disease. Lancet. 2016;388(10043):505–517. 10.1016/S0140-6736(15)01124-1 26921134

[7] Bakota L, Brandt R. Tau Biology and Tau-Directed Therapies for Alzheimer's Disease. Drugs. 2016;76(3):301–313. 10.1007/s40265-015-0529-0 26729186 PMC4757605

[8] Farooqui AA. Lipid mediators and their metabolism in the nucleous: implications for Alzheimer's disease. J Alzheimers Dis. 2012;30 Suppl 2:S163–178. 10.3233/JAD-2011-111085 21955817

[9] Dietzel J, Haeusler KG, Endres M. Does atrial fibrillation cause cognitive decline and dementia? Europace. 2018;20(3):408–419. 10.1093/europace/eux031 28387847

[10] Stadler AF, Angst PD, Arce RM, Gomes SC, Oppermann RV, Susin C. Gingival crevicular fluid levels of cytokines/chemokines in chronic periodontitis: a meta-analysis. J Clin Periodontol. 2016;43(9):727–745. 10.1111/jcpe.12557 27027257

[11] Pihlstrom BL, Michalowicz BS, Johnson NW. Periodontal diseases. Lancet. 2005;366(9499):1809–1820. 10.1016/S0140-6736(05)67728-8 16298220

[12] Beck JD, Papapanou PN, Philips KH, Offenbacher S. Periodontal Medicine: 100 Years of Progress. J Dent Res. 2019;98(10):1053–1062. 10.1177/0022034519846113 31429666

[13] Kamer AR, Pirraglia E, Tsui W, Rusinek H, Vallabhajosula S, Mosconi L, et al Periodontal disease associates with higher brain amyloid load in normal elderly. Neurobiol Aging. 2015;36(2):627–633. 10.1016/j.neurobiolaging.2014.10.038 25491073 PMC4399973

[14] Sardi F, Fassina L, Venturini L, Inguscio M, Guerriero F, Rolfo E, et al Alzheimer's disease, autoimmunity and inflammation. The good, the bad and the ugly. Autoimmun Rev. 2011;11(2):149–153. 10.1016/j.autrev.2011.09.005 21996556

[15] Jiang C, Li G, Huang P, Liu Z, Zhao B. The Gut Microbiota and Alzheimer's Disease. J Alzheimers Dis. 2017;58(1):1–15. 10.3233/JAD-161141 28372330

[16] Gardener SL, Rainey-Smith SR, Martins RN. Diet and Inflammation in Alzheimer's Disease and Related Chronic Diseases: A Review. J Alzheimers Dis. 2016;50(2):301–334. 10.3233/JAD-150765 26682690

[17] Yamamoto T, Kondo K, Hirai H, Nakade M, Aida J, Hirata Y. Association between self-reported dental health status and onset of dementia: a 4-year prospective cohort study of older Japanese adults from the Aichi Gerontological Evaluation Study (AGES) Project. Psychosom Med. 2012;74(3):241–248. 10.1097/PSY.0b013e318246dffb 22408130

[18] Kaye EK, Valencia A, Baba N, Spiro A 3rd, Dietrich T, Garcia RI. Tooth loss and periodontal disease predict poor cognitive function in older men. J Am Geriatr Soc. 2010;58(4):713–718. 10.1111/j.1532-5415.2010.02788.x 20398152 PMC3649065

[19] Noble JM, Borrell LN, Papapanou PN, Elkind MS, Scarmeas N, Wright CB. Periodontitis is associated with cognitive impairment among older adults: analysis of NHANES-III. J Neurol Neurosurg Psychiatry. 2009;80(11):1206–1211. 10.1136/jnnp.2009.174029 19419981 PMC3073380

[20] Kamer AR, Morse DE, Holm-Pedersen P, Mortensen EL, Avlund K. Periodontal inflammation in relation to cognitive function in an older adult Danish population. J Alzheimers Dis. 2012;28(3):613–624. 10.3233/JAD-2011-102004 22045483

[21] Sparks Stein P, Steffen MJ, Smith C, Jicha G, Ebersole JL, Abner E, et al Serum antibodies to periodontal pathogens are a risk factor for Alzheimer's disease. Alzheimers Dement. 2012;8(3):196–203. 10.1016/j.jalz.2011.04.006 22546352 PMC3712346

[22] Chow S-C, Shao J, Wang H, Lokhnygina Y. Sample Size Calculations in Clinical Research. 3rd ed. Boca Raton: Taylor & Francis; 2017.

[23] World Health Organization. Oral health surveys: basic methods 5th ed. Geneva: World Health Organization; 2013.

[24] WHO Scientific Group. Epidemiology, etiology and prevention of periodontal diseases Geneva: World Health Organization; 1978. 100973

[25] Gurbuz O, Alatas G, Kurt E, Dogan F, Issever H. Periodontal health and treatment needs among hospitalized chronic psychiatric patients in Istanbul, Turkey. Community Dent Health. 2011;28(1):69–74. 21485239

[26] Preshaw PM. Detection and diagnosis of periodontal conditions amenable to prevention. BMC Oral Health. 2015;15 Suppl 1:S5 10.1186/1472-6831-15-S1-S5 26390822 PMC4580822

[27] Petersen PE, Ogawa H. Strengthening the prevention of periodontal disease: the WHO approach. J Periodontol. 2005;76(12):2187–2193. 10.1902/jop.2005.76.12.2187 16332229

[28] Mehlotra RK, Hall NB, Willie B, Stein CM, Weinberg A, Zimmerman PA, et al Associations of Toll-Like Receptor and β-Defensin Polymorphisms with Measures of Periodontal Disease (PD) in HIV+ North American Adults: An Exploratory Study. PLoS One. 2016;11(10):e0164075 10.1371/journal.pone.0164075 27727278 PMC5058471

[29] Petersen PE, Ogawa H. The global burden of periodontal disease: towards integration with chronic disease prevention and control. Periodontol 2000. 2012;60(1):15–39. 10.1111/j.1600-0757.2011.00425.x 22909104

[30] Su CW, Yen AF, Lai H, Lee Y, Chen HH, Chen SS. Effects of risk factors on periodontal disease defined by calibrated community periodontal index and loss of attachment scores. Oral Dis. 2017;23(7):949–955. 10.1111/odi.12678 28419664 PMC5599987

[31] Royzman D, Recio L, Badovinac RL, Fiorellini J, Goodson M, Howell H, et al The effect of aspirin intake on bleeding on probing in patients with gingivitis. J Periodontol. 2004;75(5):679–684. 10.1902/jop.2004.75.5.679 15212350

[32] Suzuki J, Imai Y, Aoki M, Fujita D, Aoyama N, Tada Y, et al Periodontitis in cardiovascular disease patients with or without Marfan syndrome—a possible role of Prevotella intermedia. PLoS One. 2014;9(4):e95521 10.1371/journal.pone.0095521 24748407 PMC3991676

[33] Lang NP, Joss A, Orsanic T, Gusberti FA, Siegrist BE. Bleeding on probing. A predictor for the progression of periodontal disease? J Clin Periodontol. 1986;13(6):590–596. 10.1111/j.1600-051x.1986.tb00852.x 3489010

[34] Darout IA, Albandar JM, Skaug N. Periodontal status of adult Sudanese habitual users of miswak chewing sticks or toothbrushes. Acta Odontol Scand. 2000;58(1):25–30. 10.1080/000163500429398 10809396

[35] Irigoyen-Camacho ME, Sanchez-Perez L, Molina-Frechero N, Velazquez-Alva C, Zepeda-Zepeda M, Borges-Yanez A. The relationship between body mass index and body fat percentage and periodontal status in Mexican adolescents. Acta Odontol Scand. 2014;72(1):48–57. 10.3109/00016357.2013.797100 23692334

[36] Petersen RC, Stevens JC, Ganguli M, Tangalos EG, Cummings JL, DeKosky ST. Practice parameter: early detection of dementia: mild cognitive impairment (an evidence-based review). Report of the Quality Standards Subcommittee of the American Academy of Neurology. Neurology. 2001;56(9):1133–1142. 10.1212/wnl.56.9.1133 11342677

[37] Nie J, Luo Y, Huang XN, Gong QH, Wu Q, Shi JS. Icariin inhibits beta-amyloid peptide segment 25–35 induced expression of beta-secretase in rat hippocampus. Eur J Pharmacol. 2010;626(2–3):213–218. 10.1016/j.ejphar.2009.09.039 19782061

[38] Vorhees CV, Williams MT. Morris water maze: procedures for assessing spatial and related forms of learning and memory. Nat Protoc. 2006;1(2):848–858. 10.1038/nprot.2006.116 17406317 PMC2895266

[39] Gong QH, Wang Q, Pan LL, Liu XH, Huang H, Zhu YZ. Hydrogen sulfide attenuates lipopolysaccharide-induced cognitive impairment: a pro-inflammatory pathway in rats. Pharmacol Biochem Behav. 2010;96(1):52–58. 10.1016/j.pbb.2010.04.006 20399805

[40] Cao Y, Xiao Y, Ravid R, Guan ZZ. Changed clathrin regulatory proteins in the brains of Alzheimer's disease patients and animal models. J Alzheimers Dis. 2010;22(1):329–342. 10.3233/JAD-2010-100162 20847448

[41] Kujawski S, Kujawska A, Gajos M, Topka W, Perkowski R, Androsiuk-Perkowska J, et al Cognitive Functioning in Older People. Results of the First Wave of Cognition of Older People, Education, Recreational Activities, Nutrition, Comorbidities, Functional Capacity Studies (COPERNICUS). Front Aging Neurosci. 2018;10:421 10.3389/fnagi.2018.00421 30622469 PMC6308301

[42] Ren L, Bai L, Wu Y, Ni J, Shi M, Lu H, et al Prevalence of and Risk Factors for Cognitive Impairment Among Elderly Without Cardio- and Cerebrovascular Diseases: A Population-Based Study in Rural China. Front Aging Neurosci. 2018;10:62 10.3389/fnagi.2018.00062 29643801 PMC5882828

[43] Schober P, Boer C, Schwarte LA. Correlation Coefficients: Appropriate Use and Interpretation. Anesth Analg. 2018;126(5):1763–1768. 10.1213/ANE.0000000000002864 29481436

[44] Hoo ZH, Candlish J, Teare D. What is an ROC curve? Emerg Med J. 2017;34(6):357–359. 10.1136/emermed-2017-206735 28302644

[45] Ciesielska N, Sokolowski R, Mazur E, Podhorecka M, Polak-Szabela A, Kedziora-Kornatowska K. Is the Montreal Cognitive Assessment (MoCA) test better suited than the Mini-Mental State Examination (MMSE) in mild cognitive impairment (MCI) detection among people aged over 60? Meta-analysis. Psychiatr Pol. 2016;50(5):1039–1052. 10.12740/PP/45368 27992895

[46] Tombaugh TN, McIntyre NJ. The mini-mental state examination: a comprehensive review. J Am Geriatr Soc. 1992;40(9):922–935. 10.1111/j.1532-5415.1992.tb01992.x 1512391

[47] Arevalo-Rodriguez I, Smailagic N, Roqué I Figuls M, Ciapponi A, Sanchez-Perez E, Giannakou A, et al Mini-Mental State Examination (MMSE) for the detection of Alzheimer's disease and other dementias in people with mild cognitive impairment (MCI). Cochrane Database Syst Rev. 2015;2015(3):CD010783 10.1002/14651858.CD010783.pub2 25740785 PMC6464748

[48] Seraj Z, Al-Najjar D, Akl M, Aladle N, Altijani Y, Zaki A, et al The Effect of Number of Teeth and Chewing Ability on Cognitive Function of Elderly in UAE: A Pilot Study. Int J Dent. 2017;2017:5732748 10.1155/2017/5732748 29348749 PMC5734010

[49] Kim EK, Lee SK, Choi YH, Tanaka M, Hirotsu K, Kim HC, et al Relationship between chewing ability and cognitive impairment in the rural elderly. Arch Gerontol Geriatr. 2017;70:209–213. 10.1016/j.archger.2017.02.006 28214402

[50] Valero J, Bernardino L, Cardoso FL, Silva AP, Fontes-Ribeiro C, Ambrósio AF, et al Impact of Neuroinflammation on Hippocampal Neurogenesis: Relevance to Aging and Alzheimer's Disease. J Alzheimers Dis. 2017;60(s1):S161–S168. 10.3233/JAD-170239 28671124

[51] Liu C, Feng X, Li Q, Wang Y, Li Q, Hua M. Adiponectin, TNF-α and inflammatory cytokines and risk of type 2 diabetes: A systematic review and meta-analysis. Cytokine. 2016;86:100–109. 10.1016/j.cyto.2016.06.028 27498215

[52] Decourt B, Lahiri DK, Sabbagh MN. Targeting Tumor Necrosis Factor Alpha for Alzheimer's Disease. Curr Alzheimer Res. 2017;14(4):412–425. 10.2174/1567205013666160930110551 27697064 PMC5328927

[53] Gaur S, Agnihotri R. Alzheimer's disease and chronic periodontitis: is there an association? Geriatr Gerontol Int. 2015;15(4):391–404. 10.1111/ggi.12425 25511390

[54] Dominy SS, Lynch C, Ermini F, Benedyk M, Marczyk A, Konradi A, et al *Porphyromonas gingivalis* in Alzheimer's disease brains: Evidence for disease causation and treatment with small-molecule inhibitors. Sci Adv. 2019;5(1):eaau3333 10.1126/sciadv.aau3333 30746447 PMC6357742

[55] Harding A, Robinson S, Crean S, Singhrao SK. Can Better Management of Periodontal Disease Delay the Onset and Progression of Alzheimer's Disease? J Alzheimers Dis. 2017;58(2):337–348. 10.3233/JAD-170046 28453484

