# Supplementary material for: Poor oral health conditions and cognitive decline: Studies in humans and rats
Source: PLoS One. 2020 Jul 2;15(7):e0234659. doi: 10.1371/journal.pone.0234659 (PMC7332063; doi:10.1371/journal.pone.0234659)
Supplement: S1 Checklist — (DOCX) [file pone.0234659.s001.docx]

STROBE Statement—checklist of items that should be included in reports of observational studies

|  | Item No. | Recommendation | Page  No. | Relevant text from manuscript |
| --- | --- | --- | --- | --- |
| **Title and abstract** | 1 | (*a*) Indicate the study’s design with a commonly used term in the title or the abstract | 2 | cross-sectional study |
|  |  | (*b*) Provide in the abstract an informative and balanced summary of what was done and what was found | 2 | Methods and results |
| Introduction | | | |  |
| Background/rationale | 2 | Explain the scientific background and rationale for the investigation being reported | 3 | From line 44 to line 66 |
| Objectives | 3 | State specific objectives, including any prespecified hypotheses | 4 | we hypothesized that poor oral hygiene correlates with, and may even lead to, cognitive decline |
| Methods | | | |  |
| Study design | 4 | Present key elements of study design early in the paper | 4,5, 8,9 | Study population, Dental examinations, Cognitive testing, Ethics statement, Statistical analyses |
| Setting | 5 | Describe the setting, locations, and relevant dates, including periods of recruitment, exposure, follow-up, and data collection | 4 | Study population |
| Participants | 6 | (*a*) *Cohort study*—Give the eligibility criteria, and the sources and methods of selection of participants. Describe methods of follow-up  *Case-control study*—Give the eligibility criteria, and the sources and methods of case ascertainment and control selection. Give the rationale for the choice of cases and controls  *Cross-sectional study*—Give the eligibility criteria, and the sources and methods of selection of participants | 4 | Study population |
|  |  | (*b*) *Cohort study*—For matched studies, give matching criteria and number of exposed and unexposed  *Case-control study*—For matched studies, give matching criteria and the number of controls per case | N/A | N/A |
| Variables | 7 | Clearly define all outcomes, exposures, predictors, potential confounders, and effect modifiers. Give diagnostic criteria, if applicable | 4,5, 8,9 | Study population, Dental examinations, Cognitive testing, Ethics statement, Statistical analyses |
| Data sources/ measurement | 8* | For each variable of interest, give sources of data and details of methods of assessment (measurement). Describe comparability of assessment methods if there is more than one group | 4,5, 8,9 | Study population, Dental examinations, Cognitive testing, Ethics statement, Statistical analyses |
| Bias | 9 | Describe any efforts to address potential sources of bias | 9, 12 | Statistical analyses. **Table 2.** Participants’ information by medical history and smoking |
| Study size | 10 | Explain how the study size was arrived at | 4 | 102 individuals |

Continued on next page

| Quantitative variables | 11 | Explain how quantitative variables were handled in the analyses. If applicable, describe which groupings were chosen and why | 9 | We used multiple linear regression and stepwise method to analyze the relationship between MMSE scores and potential factors. |
| --- | --- | --- | --- | --- |
| Statistical methods | 12 | (*a*) Describe all statistical methods, including those used to control for confounding | 9 | A two-tailed probability of < 0.05 was considered significant for all tests. Descriptive statistics for certain characteristics were calculated and these data are shown as the number of cases (n [%]) and mean (95% CI). Independent sample t-tests were used to compare the significance of differences between continuous variable means when only two groups were compared. Continuous variable means of more than two groups were compared using a one-way analysis of variance (ANOVA). We used multiple linear regression and stepwise method to analyze the relationship between MMSE scores and potential factors. Based on past researches [27, 28], we selected the following independent variables: gender, age (years), education level, hypertension, diabetes, cardiovascular diseases, hyperlipidemia, prior stroke events, cerebral trauma, smoke, number of missing index teeth, and grouped number of missing index teeth of 0-6 and 7-10. The dependent variable was MMSE score. Spearman rank correlation was used to analyze monotonic associations for ordinal data. |
|  |  | (*b*) Describe any methods used to examine subgroups and interactions | 9 | We used multiple linear regression and stepwise method to analyze the relationship between MMSE scores and potential factors. |
|  |  | (*c*) Explain how missing data were addressed | No missing data |  |
|  |  | (*d*) *Cohort study*—If applicable, explain how loss to follow-up was addressed  *Case-control study*—If applicable, explain how matching of cases and controls was addressed  *Cross-sectional study*—If applicable, describe analytical methods taking account of sampling strategy | 4 | *Cross-sectional study*—in Nanjing, 102 individuals 52 to 101 years old were enrolled. The participants were invited to participate in questionnaire surveys and dental examinations. |
|  |  | (*e*) Describe any sensitivity analyses | No sensitivity analyses |  |
| Results | | | | |
| Participants | 13* | (a) Report numbers of individuals at each stage of study—eg numbers potentially eligible, examined for eligibility, confirmed eligible, included in the study, completing follow-up, and analysed | 9 | The final results for humans are based on 102 individuals (69 female) and 1,020 teeth (514 missing). |
|  |  | (b) Give reasons for non-participation at each stage | No non-participation |  |
|  |  | (c) Consider use of a flow diagram | 102 individuals from beginning to end |  |
| Descriptive data | 14* | (a) Give characteristics of study participants (eg demographic, clinical, social) and information on exposures and potential confounders | 10-12 | **Table 1.** Participants’ information by age, gender, and education years  **Table 2.** Participants’ information by medical history and smoking |
|  |  | (b) Indicate number of participants with missing data for each variable of interest | No missing data |  |
|  |  | (c) *Cohort study*—Summarise follow-up time (eg, average and total amount) | *Cross-sectional study* |  |
| Outcome data | 15* | *Cohort study*—Report numbers of outcome events or summary measures over time |  |  |
|  |  | *Case-control study—*Report numbers in each exposure category, or summary measures of exposure |  |  |
|  |  | *Cross-sectional study—*Report numbers of outcome events or summary measures | *14-16* | **Table 3.** Dental examinations by missing index teeth, BOP^a^, and PD^b^ levels |
| Main results | 16 | (*a*) Give unadjusted estimates and, if applicable, confounder-adjusted estimates and their precision (eg, 95% confidence interval). Make clear which confounders were adjusted for and why they were included | All unadjusted estimates |  |
|  |  | (*b*) Report category boundaries when continuous variables were categorized | 13 | According to the ROC curve, we grouped the number of missing index teeth as 0-6 and 7-10 |
|  |  | (*c*) If relevant, consider translating estimates of relative risk into absolute risk for a meaningful time period | Not relevant |  |

Continued on next page

| Other analyses | 17 | Report other analyses done—eg analyses of subgroups and interactions, and sensitivity analyses | No analyses of subgroups or interactions, or sensitivity analyses |  |
| --- | --- | --- | --- | --- |
| Discussion | | | | |
| Key results | 18 | Summarise key results with reference to study objectives | 20 | the number of missing index teeth ≥ 7 was associated with lower cognitive scores. A higher number of missing index teeth was a risk factor for lower MMSE scores. There was a negative relationship between MMSE scores and average PD. |
| Limitations | 19 | Discuss limitations of the study, taking into account sources of potential bias or imprecision. Discuss both direction and magnitude of any potential bias | 22 | Several limitations of this study should be noted. First, the human participants were grouped in general categories without considering subdivisions, such as oral health, gingivitis, and periodontitis, since the sample size was relatively small. Second, for ethical reasons, we did not collect blood samples and imaging data of the volunteers. Blood samples and imaging would allow a more accurate analysis of oral health conditions and cognitive state of the participants. |
| Interpretation | 20 | Give a cautious overall interpretation of results considering objectives, limitations, multiplicity of analyses, results from similar studies, and other relevant evidence | 20-23 | discussion |
| Generalisability | 21 | Discuss the generalisability (external validity) of the study results | 22 | The maintenance of oral health may be a new frontier in cognitive preservation among the aging population. Good oral hygiene, treatment of periodontitis, and maintenance of overall oral health may all be useful treatment strategies for the conservation of optimal neuronal function. This approach may advance the understanding, prevention, and management of aging-associated conditions. Non-pharmaceutical interventions, such as proper brushing and flossing, cleaning teeth regularly, curing periodontitis, and maintaining teeth are relatively simple, inexpensive and non-invasive approaches for delaying cognitive decline and AD. |
| Other information | |  | | |
| Funding | 22 | Give the source of funding and the role of the funders for the present study and, if applicable, for the original study on which the present article is based | 23 | This work was supported by the China Oral Health Foundation (Grant No. 2016037 to SZ), Jiangsu Provincial Medical Innovation Team, the Project of Invigorating Health Care through Science, Technology and Education (Grant No. CXTDB2017014 to FuY) and the National Natural Science Foundation of China (Grant Nos. 81260168 and 81860197 to SG). |

*Give information separately for cases and controls in case-control studies and, if applicable, for exposed and unexposed groups in cohort and cross-sectional studies.

**Note:** An Explanation and Elaboration article discusses each checklist item and gives methodological background and published examples of transparent reporting. The STROBE checklist is best used in conjunction with this article (freely available on the Web sites of PLoS Medicine at http://www.plosmedicine.org/, Annals of Internal Medicine at http://www.annals.org/, and Epidemiology at http://www.epidem.com/). Information on the STROBE Initiative is available at www.strobe-statement.org.
