# Supplementary figures and images for: Poor oral health conditions and cognitive decline: Studies in humans and rats
Source: PLoS One. 2020 Jul 2;15(7):e0234659. doi: 10.1371/journal.pone.0234659 (PMC7332063; doi:10.1371/journal.pone.0234659)

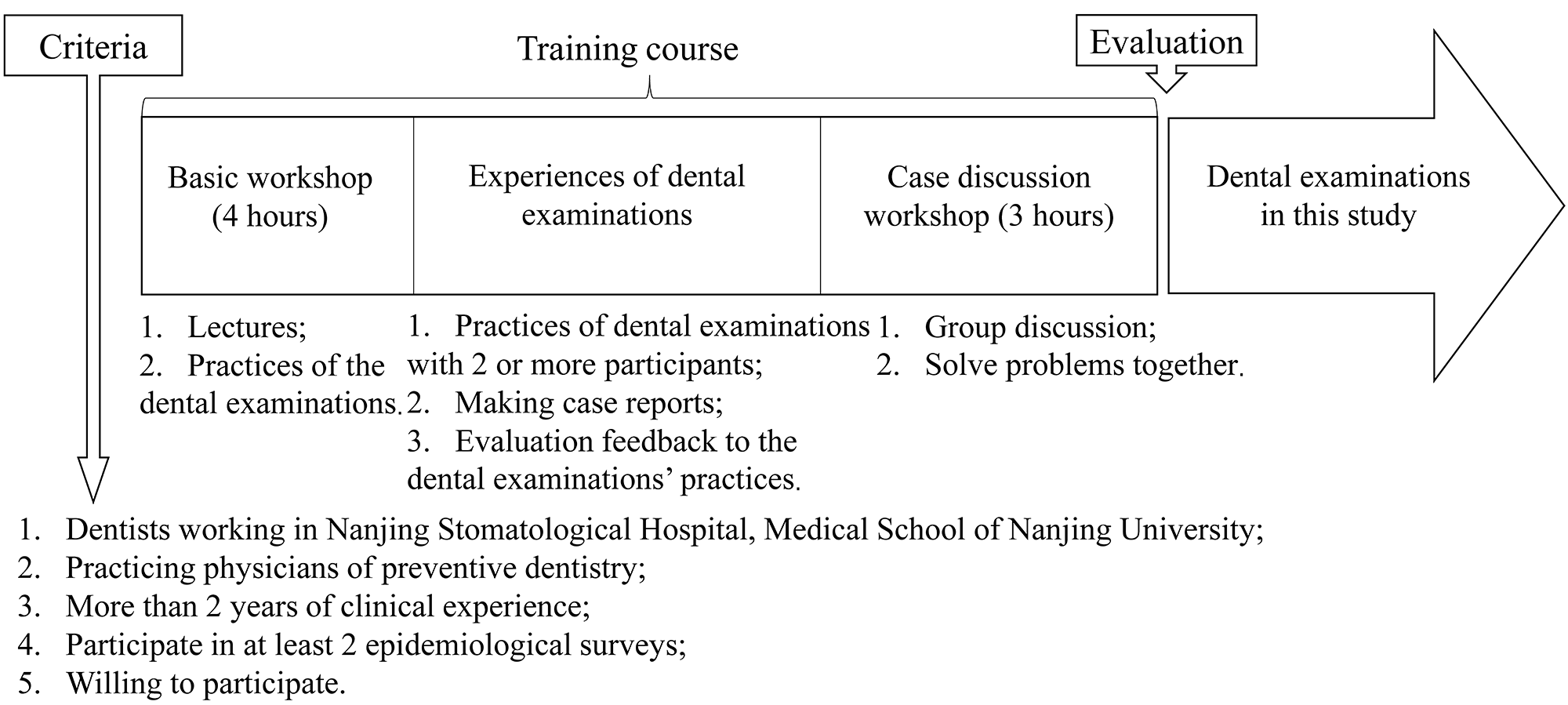

Supplement: S1 Fig — (TIF) [file pone.0234659.s002.tif]

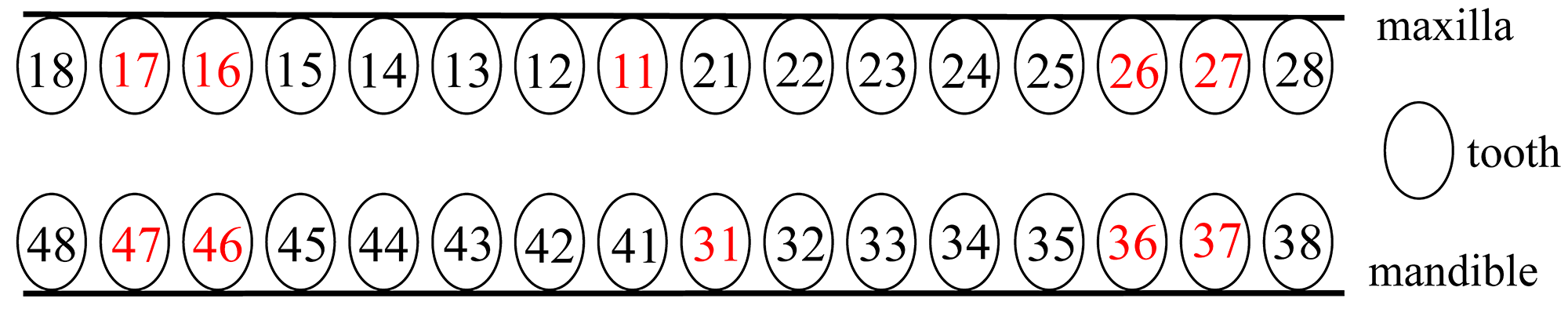

Supplement: S2 Fig — The numbers assigned to teeth are their specified names in dentistry. The 10 teeth labelled in red are the index teeth according to the Community Periodontal Index. (TIF) [file pone.0234659.s003.tif]
